# Comparison of Biliary Complications Between Living and Deceased Donor Liver Transplantations: A Systematic Review and Meta-analysis

**DOI:** 10.7759/cureus.69019

**Published:** 2024-09-09

**Authors:** Suprabhat Giri, Saroj K Sahu, Vedavyas Mohapatra, Mansi Chaudhary, Manas Panigrahi, Preetam Nath, Bipadabhanjan Mallick, Dibya L Praharaj

**Affiliations:** 1 Gastroenterology and Hepatology, Kalinga Institute of Medical Sciences, Bhubaneswar, IND; 2 Surgical Gastroenterology, Kalinga Institute of Medical Sciences, Bhubaneswar, IND; 3 Gastroenterology, All India Institute of Medical Sciences, Bhubaneswar, IND

**Keywords:** bile leak, deceased donor liver transplantation, liver transplantation, living donor liver transplantation, post-transplant biliary stricture

## Abstract

To understand if the risk of biliary complications is higher with living donor liver transplantation (LDLT) compared to deceased donor liver transplantation (DDLT), the present meta-analysis was conducted to analyze the differences between these two types of liver transplantations. Three databases were searched from inception to September 2023 for comparative studies reporting biliary complications with LDLT and DDLT. Odds ratios (OR) with 95% confidence intervals were calculated for all the dichotomous outcomes. Twenty-eight studies were included in the final analysis. LDLT was associated with a significantly higher incidence of biliary complications than DDLT (OR 1.96, 95% CI: 1.56-2.47). However, on subgroup analysis, only studies published in or before 2014 reported a higher incidence of biliary complications with LDLT, but not with studies published after 2014. An analysis of individual adverse events showed that LDLT was associated with a higher incidence of both bile leak (OR 3.38, 95% CI: 2.52-4.53) and biliary stricture (OR 1.75, 95% CI: 1.20-2.55). LDLT was associated with a higher incidence of overall biliary complications, including bile leak and biliary stricture. With advances in surgical techniques, there has been a reduction in the risk of biliary complications.

## Introduction and background

Liver transplantation (LT) is often regarded as the definite treatment option for the management of end-stage liver disease (ESLD), acute liver failure (ALF), and primary liver cancers. With advances in surgical techniques and immunosuppression regimens, there has been a remarkable improvement in the survival rates of these patients. At present, the reported five-year survival following a successful LT stands at 70-75% [1,2].

Following LT, biliary complications are common and constitute a significant cause of morbidity and mortality. Up to 25% of patients may develop biliary complications after undergoing LT, out of which 10% may ultimately die of these complications. Common biliary complications after LT include biliary stricture (both non-anastomotic and anastomotic) and bile leak. Rare complications include bile stones, clots, bile cast syndrome, and hemobilia [3]. Timely diagnosis and management are necessary to salvage the graft and improve long-term outcomes [4]. In India and most Southeast Asian countries, live donor liver transplant (LDLT) constitutes the major bulk of LT in contrast to Western countries due to the lower rate of cadaveric organ donation [5].

Traditionally, biliary complications were thought to be higher in LDLT than in DDLT. The reported biliary complications following LDLT are about two to three times higher than those with cadaveric LT. Multiple factors related to surgical techniques are major reasons for increased biliary complications. Extensive hilar dissection in LDLT leads to disturbed blood supply to bile ducts and ultimately causes various biliary complications in these patients [6]. However, a better understanding of vascular anatomy and improved surgical techniques have significantly reduced these complications, even in LDLT settings. However, an updated systematic review and meta-analysis are lacking in this regard. The primary aim of this systematic review and meta-analysis is to provide updated data regarding the incidence of various biliary complications in the setting of LDLT in contrast to cadaveric LT.

## Review

Methods

Information Sources and Search Strategy

A comprehensive search was conducted using the databases of MEDLINE, EMBASE, and Scopus from inception to September 2023. The keywords used were: (Liver OR Hepat* OR HCC OR Cirrhosis) AND (LDLT OR Live donor OR Living donor) AND (DDLT OR Deceased donor OR Cadaveric) AND (Biliary OR Bile OR Bile duct AND (Complication OR Adverse events OR Leak OR Stricture). The manual searching of reference lists of the included studies was also undertaken to ensure that all potentially relevant studies were included. The systematic review and meta-analysis were conducted per the Preferred Reporting Items for Systematic Review and Meta-Analyses (PRISMA) guidelines [7].

Study Selection

All prospective and retrospective studies fulfilling the following PICO criteria were included: (a) Patients - patients with cirrhosis of the liver undergoing LT; (b) Intervention - LDLT; (c) Comparison - DDLT; (d) Outcomes - biliary complications. The biliary complications included both bile leak and stricture. In accordance with the selection criteria above, the titles and abstracts of all studies were independently reviewed by two authors. A third reviewer resolved any disagreements. The exclusion criteria used were non-comparative studies, conference abstracts, case series, and non-English studies.

Data Extraction and Quality Assessment

Two reviewers independently extracted the data, while a third reviewer arbitrated any conflicts. Each study's title, first author, year of publication, country, number of patients, age and sex distribution, indication for TIPS, outcome metrics, and follow-up time were all listed on the form. Using a Newcastle-Ottawa scale for cohort studies [8], two independent reviewers evaluated the quality of the included studies. In the event of a disagreement, a third reviewer was contacted.

Statistical Analysis

Odds ratios (OR) with 95% confidence intervals (CI) were calculated for all the dichotomous outcomes. Regardless of heterogeneity, the Mantel-Haenszel test for random effects was used. Cochran's Q test and I^2^ statistics were used to determine the heterogeneity between the studies. A P-value of Q test < 0.1 or the I^2^ value > 30% was significant. Publication bias was assessed by visual inspection of the funnel plot. Subgroup analysis and leave-one-out meta-analysis were conducted for sensitivity analysis. RevMan software (version 5.4.1, Cochrane Collaboration) and STATA software (version 17, StataCorp., College Station, TX) were used for statistical analysis.

Results

Baseline Characteristics and Quality Assessment of Included Studies

**Figure 1:**
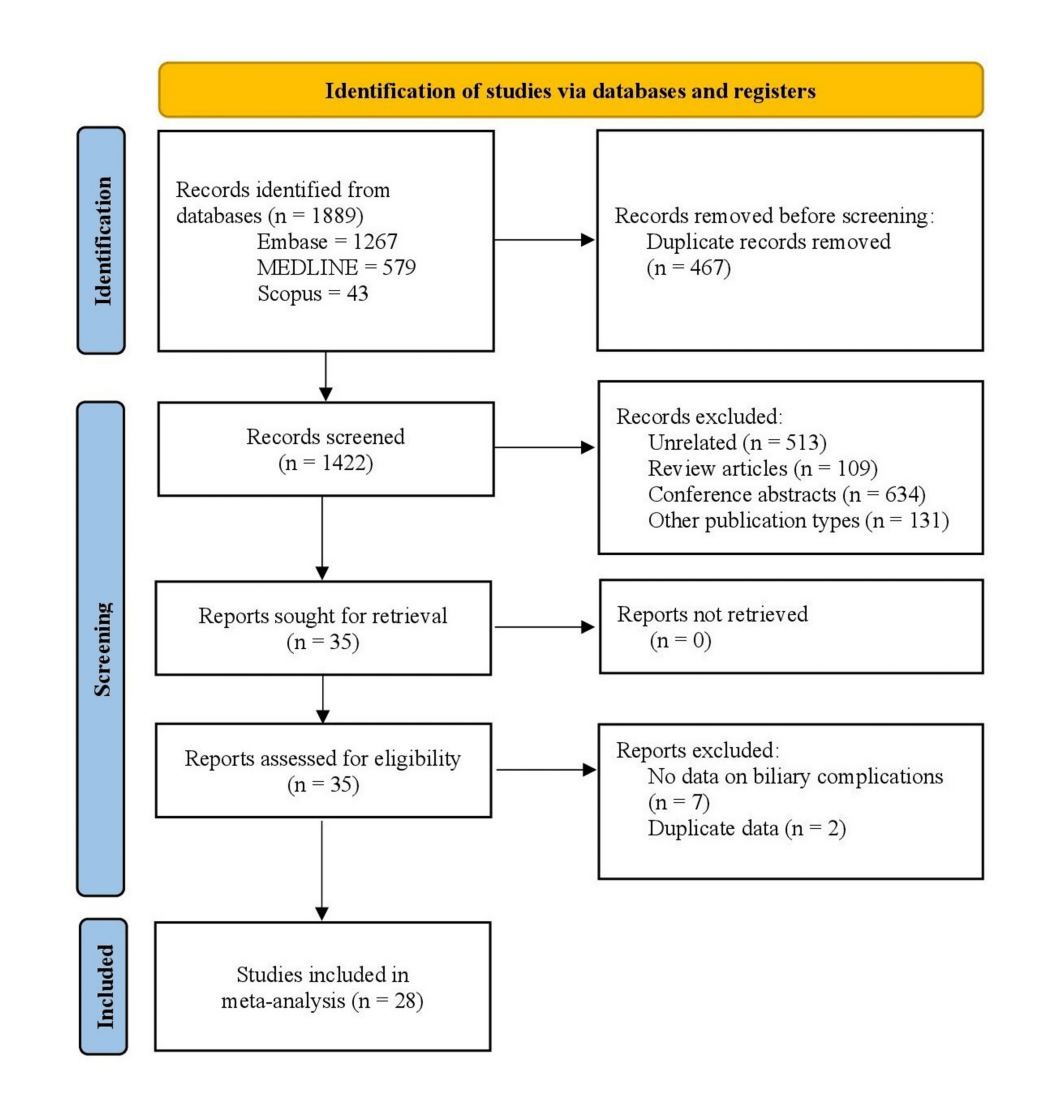
PRISMA flowchart showing the study identification, selection, and inclusion process. PRISMA: Preferred Reporting Items for Systematic Review and Meta-Analyses

The above search strategy yielded 1889 records, out of which 28 studies were included in the final analysis [9-36]. Figure 1 shows the flowchart for the study selection and inclusion process. Tables 1, 2 summarize the baseline characteristics and outcomes of individual studies included in the meta-analysis. All the studies were retrospective in nature except for the one by Liu et al. [10]. Fourteen studies were from Asian countries [10,11,16,17,19,20,23,24,26,29-31,34,36], 11 were from North America [12-14,18,21,22,25,27,28,32,33], and one each from Europe [9] and South America [15]. Two studies included some pediatric patients [11,17], while the rest included adult patients exclusively. Three studies included patients with hepatocellular carcinoma (HCC) exclusively [20,24,26]. Eleven studies were of good quality [9,10,16,19-21,24,25,27,29,36], 13 studies were of fair quality, [11-14,17,22,23,26,28,30,32,33,35], and four were of poor quality [15,18,31,34].

**Table 1:** Baseline characteristics of the studies included in the meta-analysis DDLT: Deceased donor liver transplantation; HCC: Hepatocellular carcinoma; LDLT: Living donor liver transplantation; MELD: Model for End-Stage Liver Disease

Author, year	Country, No. of centers	Design	Arm	No. of patients	Age, in years	Male sex	MELD score	Patients with HCC	Study quality
Garcia-Retortillo 2004 [9]	Spain, Single	Retrospective	LDLT	22	59 (24–68)	13 (59.1%)	11 (5–24)	13 (59.1%)	Good
			DDLT	95	59 (38–66)	58 (61.1%)	11 (2–28)	45 (47.4%)	
Liu et al. 2006 [10]	Hong Kong, Single	Prospective	LDLT	124	47.5 (18–68)	97 (78.2%)	21 (7–46)	36 (29%)	Good
			DDLT	56	48 (27–66)	44 (78.6%)	19 (6–49)	11 (19.6%)	
Al-Sebayel et al. 2007 [11]	Saudi Arabia, Single	Retrospective	LDLT	45	47 (1.5–63)	29 (64.4%)	-	21 (17%)	Fair
			DDLT	77	44 (11–63)	38 (49.3%)	-		
Freise et al. 2008 [12]	USA, Multicentric	Retrospective	LDLT	384	49.6 ± 10.7	222 (58%)	15 ± 6	63 (16%)	Fair
			DDLT	216	51.4 ± 9.7	128 (59%)	21 ± 9	39 (18%)	
Lai et al. 2009 [13]	USA, Single	Retrospective	LDLT	86	50.6 ± 12.2	42 (49%)	20.5 ± 5.1	31 (36%)	Fair
			DDLT	403	53.6 ± 10.8	289 (72%)	23.0 ± 9.8	126 (31%)	
Fisher et al. 2009 [14]	USA, Single	Retrospective	LDLT	107	48.5 ± 12.0	66 (61.7%)	14.1 ± 0.6	8 (7.4%)	Fair
			DDLT	465	51.5 ± 8.6	366 (78.7%)	18.7 ± 0.4	134 (28.9%)	
Gómez et al. 2009 [15]	Argentina, Single	Retrospective	LDLT	30	-	-	-	-	Poor
			DDLT	357	-	-	-	-	
Li et al. 2011 [16]	China, Single	Retrospective	LDLT	128	42.96 ± 8.57	108	19.5 ± 10.7	0	Good
			DDLT	221	44.55 ± 9.71	179	18.2 ± 9.6	0	
Saha et al. 2012 [17]	India, Single	Retrospective	LDLT	18	21.6 (0.5–61)	-	-	0	Fair
			DDLT	35	35.2 (1.2–63)	-	-	0	
Chan et al. 2013 [18]	Canada, Single	Retrospective	LDLT	29	-	-	-	-	Poor
			DDLT	333	-	-	-	-	
Jiang et al. 2013 [19]	China, Single	Retrospective	LDLT	70	40.2 ± 8.1	62 (88.6%)	23.9 ± 5.6	0	Good
			DDLT	191	44.1 ± 9.3	162 (84.8%)	21.7 ± 5.7	0	
Lei et al. 2013 [20]	China, Single	Retrospective	LDLT	31	44.4 ± 9.7	18 (58.1%)	9.3 ± 6.1	31 (100%)	Good
			DDLT	52	44.0 ± 8.2	31 (59.6%)	9.1 ± 5.8	52 (100%)	
Reichman et al. 2013 [21]	Canada, Single	Retrospective	LDLT	145	54.2 ± 7.5	117 (80.7%)	14.4 (6–29)	55 (37.9%)	Good
			DDLT	145	53.9 ± 7.7	117 (80.7%)	14 (6–33)	80 (55%)	
Zimmerman et al. 2013 [22]	USA, Multicentric	Retrospective	LDLT	356	-	-	-	-	Fair
			DDLT	189	-	-	-	-	
Kim et al. 2014 [23]	South Korea, Single	Retrospective	LDLT	21	53.1 ± 10.3	14	13.1 ± 5.4	17 (80.9%)	Fair
			DDLT	29	51.3 ± 9.2	15	24.9 ± 11.6	11 (37.9%)	
Wan et al. 2014 [24]	China, Single	Retrospective	LDLT	40	48.6 ± 9.7	34 (85%)	6-19: 87.5%	40 (100%)	Good
			DDLT	80	49.5 ± 8.9	68 (85%)	6-19: 88.7%	80 (100%)	
Sandal et al. 2015 [25]	USA, Single	Retrospective	LDLT	62	52.9 ± 9.4	36 (58.1%)	13.9 ± 4.2	2 (3.2%)	Good
			DDLT	108	52.0 ± 10.6	76 (70.4%)	20.1 ± 8.8	0	
Hu et al. 2016 [26]	China, Multicenter	Retrospective	LDLT	389	48.0 ± 8.6	360 (92.5%)	-	389 (100%)	Fair
			DDLT	6471	50.1 ± 9.4	5817 (89.9%)	-	6471 (100%)	
Samstein et al. 2016 [27]	USA, Multicentric	Retrospective	LDLT	565	51.0 ± 10.9	311 (55%)	6-15: 57%	70 (12%)	Good
			DDLT	471	52.2 ± 10.4	285 (61%)	6-15: 34%	103 (22%)	
Barbas et al. 2017 [28]	Canada, Multicenter	Retrospective	LDLT	48	54.7 ± 9.4	35 (72.9%)	17.8 ± 8.7	8 (16.7%)	Fair
			DDLT	128	56.7 ± 9.3	87 (68.0%)	21.8 ± 10.3	42 (32.8%)	
Chok et al. 2017 [29]	China, Single	Retrospective	LDLT	54	51 (19–67)	42 (77.8%)	40 (35–40)	3 (5.5%)	Good
			DDLT	40	51 (23–66)	34 (85%)	39 (35–40)	1 (2.5%)	
Kim et al. 2017 [30]	South Korea, Single	Retrospective	LDLT	109	52.0 ± 8.5	81 (74.3%)	12.5 ± 8.3	68 (62.4%)	Fair
			DDLT	76	53.1 ± 11.0	50 (65.8%)	24.9 ± 11.7	16 (21.1%)	
Miyagi et al. 2017 [31]	Japan, Single	Retrospective	LDLT	168	-	-	-	-	Poor
			DDLT	441	-	-	-	-	
Humar et al. 2019 [32]	USA, Single	Retrospective	LDLT	245	56	144 (59%)	16	54 (22%)	Fair
			DDLT	592	56	414 (70%)	22	213 (36%)	
Amara et al. 2022 [33]	USA, Multicenter	Retrospective	LDLT	109	-	57 (52.3%)	-	17 (15.6%)	Fair
			DDLT	1684	-	1135 (67.4%)	-	561 (33.3%)	
Karakaya et al. 2022 [34]	Turkey, Single	Retrospective	LDLT	151	-	-	-	-	Poor
			DDLT	23	-	-	-	-	
Meier et al. 2022 [35]	UNOS database	Retrospective	LDLT	318	53.9 ± 11.1	158 (49.7%)	35.6 ± 7.0	50 (15.7%)	Fair
			DDLT	3165	53.5 ± 10.6	2045 (64.6%)	19.0 ± 9.7	626 (19.8%)	
Lapisatepun et al. 2023 [36]	Thailand, Multicenter	Retrospective	LDLT	20	54.7 ± 11.7	14 (70%)	14.5 (12−23.5)	11 (55.0%)	Good
			DDLT	20	48.8 ± 14.3	14 (70%)	14.5 (7.5−22.5)	14 (70.0%)	

**Table 2:** Outcome of individual studies DDLT: Deceased donor liver transplantation; LDLT: Living donor liver transplantation

Author, year	Arm	No. of patients	Biliary complications	Bile leak	Biliary stricture	Anastomotic leak	Anastomotic stricture
Garcia-Retortillo 2004 [9]	LDLT	22	16 (72.7%)	-	-	-	-
	DDLT	95	21 (22.1%)	-	-	-	-
Liu et al. 2006 [10]	LDLT	124	32 (25.8%)	5 (4.0%)	31 (25.0%)	-	-
	DDLT	56	4 (7.1%)	2 (3.6%)	3 (5.4%)	-	-
Al-Sebayel et al. 2007 [11]	LDLT	45	11 (24.4%)	-	-	-	-
	DDLT	77	2 (2.5%)	-	-	-	-
Freise et al. 2008 [12]	LDLT	384	161 (41.9%)	122 (31.7%)	75 (19.5%	-	-
	DDLT	216	53 (17.9%)	22 (10.1%)	35 (16.2%)	-	-
Lai et al. 2009 [13]	LDLT	86	15 (17%)	-	-	-	-
	DDLT	403	34 (8%)	-	-	-	-
Fisher et al. 2009 [14]	LDLT	107	29 (27.1%)	-	-	-	-
	DDLT	465	82 (17.6%)	-	-	-	-
Gómez et al. 2009 [15]	LDLT	30	10 (33.3%)	4 (13.3%)	10 (33.3%)	1 (33.3%)	10 (33.3%)
	DDLT	357	34 (9.5%)	6 (1.6%)	27 (7.5%)	4 (1.12%)	27 (7.5%)
Li et al. 2011 [16]	LDLT	128	19 (14.8%)	12 (9.3%)	7 (5.4%)	-	-
	DDLT	221	24 (10.8%)	3 (1.35%)	15 (6.7%)	-	-
Saha et al. 2012 [17]	LDLT	18	5 (27.7%)	3 (16.6%)	2 (11,1%)	-	-
	DDLT	35	3 (8.5%)	1 (2.8%)	2 (5.7%)	-	-
Chan et al. 2013 [18]	LDLT	29	-	2 (6.8%)	8 (27.5%	-	8 (27.5%)
	DDLT	333	-	24 (7.2%)	39 (11.7%)	-	33 (9.9%)
Jiang et al. 2013 [19]	LDLT	70	16 (22.8%)	7 (10%)	9 (12.8%)	-	-
	DDLT	191	25 (13%)	12 (6.2%)	13 (6.8%)	-	-
Lei et al. 2013 [20]	LDLT	31	-	1 (3.2%)	0	-	-
	DDLT	52	-	1 (1.9%)	1 (1.9%)	-	-
Reichman et al. 2013 [21]	LDLT	145	50 (34.4%)	26 (17.9%)	30 (20.6%)	-	-
	DDLT	145	25 (17.2%)	7 (4.82%)	18 (12.4%)	-	-
Zimmerman et al. 2013 [22]	LDLT	356	141 (25%)	95 (26.6%)	50 (14.0%)	-	-
	DDLT	189	47 (40%)	19 (10.0%)	29 (15.3%)	-	-
Kim et al. 2014 [23]	LDLT	21	2 (9.5%)	-	-	-	-
	DDLT	29	2 (6.8%)	-	-	-	-
Wan et al. 2014 [24]	LDLT	40	-	4 (10%)	7 (17.5%)	-	-
	DDLT	80	-	1 (1.2%)	5 (6.25%)	-	-
Sandal et al. 2015 [25]	LDLT	62	20 (32.3%)	-	-	-	-
	DDLT	108	42 (38.9%)	-	-	-	-
Hu et al. 2016 [26]	LDLT	389	81 (20.8%)	-	-	-	-
	DDLT	6471	721 (11.1%)	-	-	-	-
Samstein et al. 2016 [27]	LDLT	565	-	147 (26%)	181 (32%)	-	-
	DDLT	471	-	42 (9%)	99 (21%)	-	-
Barbas et al. 2017 [28]	LDLT	48	7 (14.5%)	4 (8.3%)	3 (6.25%)	-	-
	DDLT	128	6 (4.6%)	2 (1.5%)	4 (3.12%)	-	-
Chok et al. 2017 [29]	LDLT	54	2 (3.7%)	-	2 (3.7%)	-	-
	DDLT	40	1 (2.5%)	-	1 (2.5%)	-	-
Kim et al. 2017 [30]	LDLT	109	10 (9.1%)	-	-	-	-
	DDLT	76	5 (6.5%)	-	-	-	-
Miyagi et al. 2017 [31]	LDLT	168	29 (17.2%)	-	-	-	-
	DDLT	441	82 (18.5%)	-	-	-	-
Humar et al. 2019 [32]	LDLT	245	36 (14.6%)	29 (11.8%)	12 (4.89%)	-	-
	DDLT	592	110 (18.5%)	42 (7.09%)	75 (12.6%)	-	-
Amara et al. 2022 [33]	LDLT	109	34 (31.1%)	-	-	-	-
	DDLT	1684	314 (18.6%)	-	-	-	-
Karakaya et al. 2022 [34]	LDLT	151	46 (30.4%)	-	-	-	-
	DDLT	23	8 (34.7%)	-	-	-	-
Meier et al. 2022 [35]	LDLT	138	-	50 (36.2%)	66 (47.8%)	-	60 (43.4%)
	DDLT	276	-	24 (8.6%)	87 (31.5%)	-	64 (23.1%)
Lapisatepun et al. 2023 [36]	LDLT	20	8 (40%)	-	-	-	-
	DDLT	20	2 (10%)	-	-	-	-

Biliary Complications

**Figure 2:**
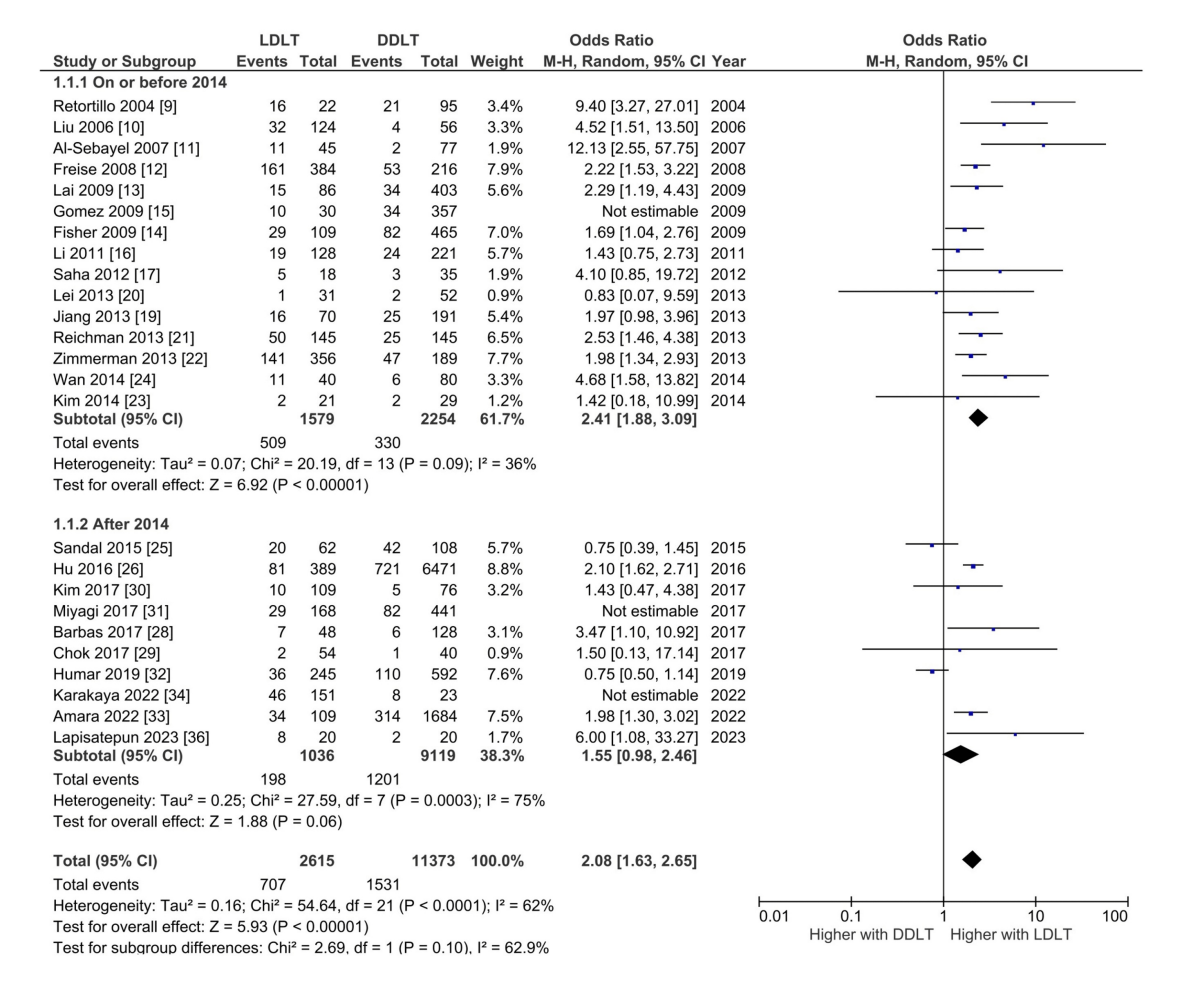
Forest plot comparing the risk of overall biliary complications between the living donor and deceased donor liver transplantation with subgroup analysis based on the year of publication. DDLT: Deceased donor liver transplantation; LDLT: Living donor liver transplantation

A total of 25 studies with 15,158 patients reported the incidence of post-LT biliary complications [9-17,19,21-23,15,26,28-34,36]. The pooled incidence of biliary complications with LDLT and DDLT were 24.4 (95% CI: 19.2-29.7; I^2^ = 92.0%) and 13.1% (95% CI: 10.6-15.5; I^2^ = 90.8%), respectively. LDLT was associated with significantly higher odds of biliary complications after LT with OR 2.00 (95% CI: 1.57-2.54; p < 0.000; I^2^ = 66%) with significant heterogeneity. On subgroup analysis of the studies based on the year of publication, with studies published on or before 2014 showing significantly higher odds of biliary complications after LT with OR 2.53 (95% CI: 1.97-3.25; p < 0.000; I^2^ = 39%) but comparable pooled odds in studies published after 2014 (OR 1.36, 95% CI: 0.92-2.02; p = 0.07; I^2^ = 74%) (Figure 2).

Bile Leak

**Figure 3:**
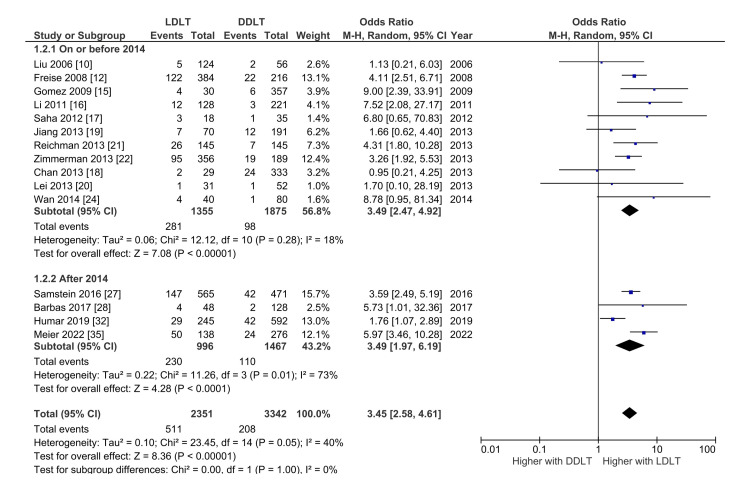
Forest plot comparing the risk of bile leak between the living donor and deceased donor liver transplantation with subgroup analysis based on the year of publication. DDLT: Deceased donor liver transplantation; LDLT: Living donor liver transplantation

Overall, 15 studies with 5693 patients analyzed the risk of bile leak between LDLT and DDLT [10,12,15-22,24,27,28,32,35]. The pooled incidence of bile leak with LDLT and DDLT were 15.5 (95% CI: 9.9 - 21.3; I^2^ = 93.1%) and 5.0% (95% CI: 3.3-6.8; I^2^ = 84.1%), respectively. LDLT was associated with significantly higher odds of biliary leak after LT with OR 3.45 (95% CI: 2.58-4.61; p < 0.000; I^2^ = 40%) without significant heterogeneity. On subgroup analysis of the studies based on the year of publication, the odds of biliary complications were higher with LDLT in both subgroups (studies published on or before 2014 and after 2014) (Figure 3).

Biliary Stricture

**Figure 4:**
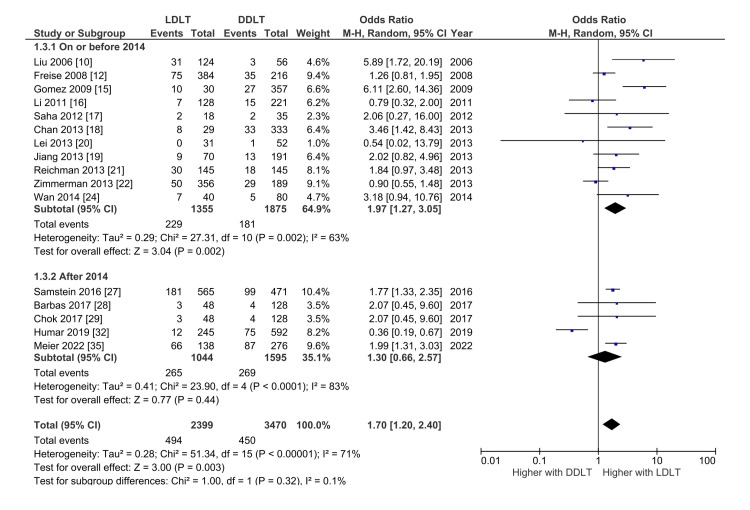
Forest plot comparing the risk of biliary stricture between the living donor and deceased donor liver transplantation with subgroup analysis based on the year of publication. DDLT: Deceased donor liver transplantation; LDLT: Living donor liver transplantation

A total of 16 studies with 5869 patients compared the risk of biliary stricture between LDLT and DDLT [10,12,15-22,24,27-29,32,35]. The pooled incidence of the biliary stricture with LDLT and DDLT were 17.2 (95% CI: 11.1-23.3; I^2^ = 94.8%) and 10.3% (95% CI: 6.9-13.6; I^2^ = 91.6%), respectively. LDLT was associated with significantly higher odds of biliary stricture after LT with OR 1.68 (95% CI: 1.19-2.39; p = 0.003; I^2^ = 71%) with significant heterogeneity. On subgroup analysis of the studies based on the year of publication, with studies published on or before 2014 showing significantly higher odds of biliary stricture after LT with OR 1.97 (95% CI: 1.27-3.05; p = 0.002; I^2^ = 63%) but comparable pooled odds in studies published after 2014 (OR 1.30, 95% CI: 0.66-2.57; p = 0.44; I^2^ = 83%) (Figure 4).

Publication Bias and Sensitivity Analysis

**Figure 5:**
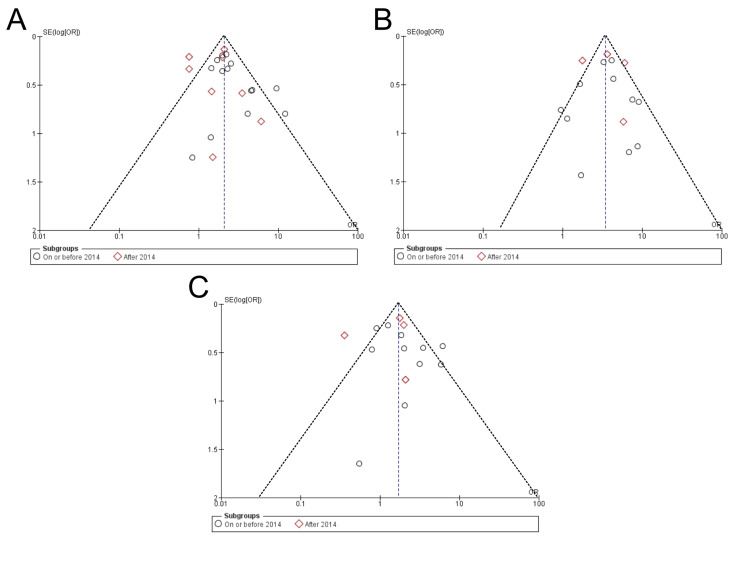
Funnel plot for assessment of publication bias with respect to (A) biliary complications, (B) bile leak, and (C) biliary stricture

Visual inspection of the funnel plot for each of the individual outcomes showed a fairly symmetrical distribution, with the majority of the dots located at the top of the plot. This indicates a higher number of studies with greater precision without any evidence of significant publication bias for any of the outcomes (Figure 5).

**Figure 6:**
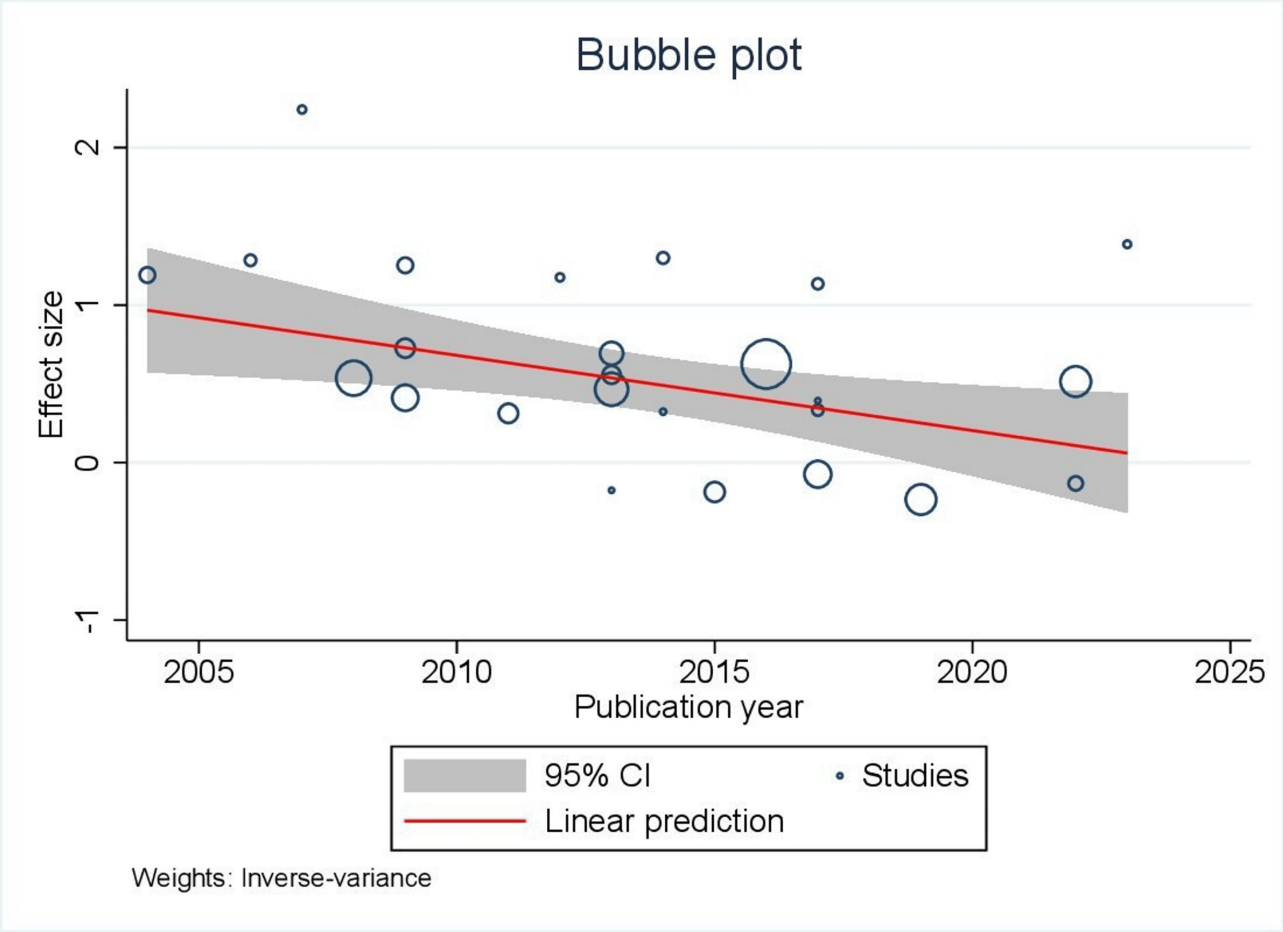
Bubble plot showing publication year as a significant covariate contributing to heterogeneity concerning biliary complication on meta-regression analysis.

Meta-regression analysis showed that publication year was a significant covariate contributing to heterogeneity concerning biliary complication (p = 0.0098) (Figure 6). This indicates that with progressing years, there was a significant reduction in the incidence of biliary complications with LT.

Leave-one-out analysis and analysis after the exclusion of poor-quality studies did not show a significant difference in the overall effect size for biliary complications or bile leaks. On the exclusion of poor-quality studies, the odds of developing a biliary stricture with LDLT reduced from 1.68 (95% CI: 1.19-2.39; I^2^ = 71%) to 1.43 (95% CI: 1.02-2.01; I^2^ = 65%).

Discussion

Traditionally, the incidence of biliary complications was thought to be higher in cases of LDLT as compared to cadaveric LT. Apart from hilar dissection in LDLT contributing to de-vascularization of the bile duct and subsequent bile leak, the need to dissect the left or right hepatic duct of the recipient increases the complexities of surgery, prolongs ischemic time, and increases the risk of biliary complications. Similarly, mobilization of the recipient hepatic duct to achieve a tension-free anastomosis may lead to disturbances in blood supply to the bile duct and consequent biliary complications [3]. Ziogas et al. compared the outcome of LDLT with donation after brain death (DBD) and donation after circulatory death (DCD) in patients with cholestatic liver disease [37]. The authors reported that the risk of graft failure was comparable between LDLT and DBD but higher with DCD, which was likely due to a high rate of biliary complications with DCD. Thus, multiple factors can predispose to a higher risk of biliary complications in LDLT.

However, with advancements in surgical techniques and immunosuppression, these complications have significantly reduced in recent times in the LDLT setting, though an updated meta-analysis is lacking at present. The primary aim of this meta-analysis was to compare incidences of various biliary complications in LDLT and DDLT settings. The present meta-analysis of 28 studies showed that around one-fourth of patients with LDLT and one-seventh of patients with DDLT develop biliary complications. LDLT was associated with a significantly higher incidence of overall biliary complications, bile leak, and biliary stricture with OR of 1.96 (1.56-2.47), 3.38 (2.52-4.53), and 1.75 (1.20-2.55), respectively. On subgroup analysis, only studies published in or before 2014 had a higher incidence of biliary complications and biliary stricture with LDLT, but not with studies published after 2014. This suggests that with improvement in surgical techniques and immunosuppression, there has been a significant reduction in the rate of biliary complications.

The most common biliary complications after LT are biliary strictures, which constitute about 50% of all cases [3]. Post-LT biliary strictures may be anastomotic (AS) or non-anastomotic (NAS). Patients undergoing LDLT are at higher risk of developing AS due to small caliber bile duct and complex anastomotic techniques followed in an LDLT procedure. All but one study included in our meta-analysis had a higher incidence of biliary strictures in the LDLT setting. In the study by Humar et al., biliovascular complications between DDLT and LDLT were comparable [32]. However, in this study, patients in the LDLT group had low Model for End-Stage Liver Disease (MELD) scores. Moreover, more patients in DDLT groups had underlying HCC. Both high MELD scores and underlying HCC have been shown to be associated with a higher risk of biliary complications [38].

In line with biliary strictures, bile leaks were also found to be higher in LDLT in most of the included studies. Like biliary strictures, bile leaks can be anastomotic or non-anastomotic. The most common type of bile leak is anastomotic, with most of the cases occurring within four weeks of LT [3]. An older review reported that the incidence of biliary stricture ranges from 5% to 15% after DDLT and 28% to 32% after LDLT [39]. In agreement with this study, our meta-analysis also found that the incidence of bile leaks continues to be higher in LDLT settings. More importantly, in a recent study by Meier et al., the incidence of bile leak was as high as 36% in LDLT in contrast to 7%-10% in the DDLT setting [35]. With respect to the timing of the development of biliary stricture, Chan et al. reported that although there was a tendency for a more delayed onset of stricture with LDLT, the mean time to stricture onset was not significantly different between the two groups (98 ±17 vs. 172±65 days, P=0.11) [18]. Zimmerman et al. also did not show any difference in the median time from transplant to onset of a biliary leak or stricture [22].

The strength of the present meta-analysis remains in the fact that the present meta-analysis included the development of biliary complications as the primary outcome, while the previous meta-analyses included it as a secondary outcome, leading to the non-inclusion of many studies. Our study, though, is an updated meta-analysis including recent studies; nevertheless, it had a few limitations. Most of the included studies were retrospective. Some of the studies also included pediatric recipients who tend to have a higher risk of biliary complications after LT, although reanalysis after exclusion of the studies did not change the risk. More importantly, the type of LT (DDLT vs. LDLT) is only one of the many risk factors for having biliary complications after transplantation (e.g., ABO-incompatible liver transplantation, cytomegalovirus infection after LT, high MELD scores, presence of underlying HCC, ischemia times and type of biliary reconstruction) [3,39]. None of these factors have been separately analyzed in our study (because of the retrospective nature of most of the studies). We could not compare the risk of anastomotic and non-anastomotic strictures separately, as the data regarding the same were not available in the majority of the studies. None of the included studies in our meta-analysis looked separately into incidences of nonanastomotic strictures (NAS) in DDLT and LDLT settings. Similarly, rare biliary complications after LT, like choledocholithiasis, bile cast, hemobilia, and sphincter of Oddi dysfunction, have not been analyzed. Lastly, there was significant heterogeneity for all the outcomes reducing the strength of evidence.

## Conclusions

To conclude, biliary complications, including biliary strictures and bile leaks, continue to be major causes of morbidity and mortality after LT. LDLT is associated with a higher incidence of biliary complications, including bile leak and biliary stricture, compared to DDLT. While improved surgical techniques and immunosuppression have reduced the incidence of biliary complications significantly, more is left to be desired. Further high-quality prospective studies are needed to provide a reliable database to compare the incidence of biliary complications between LDLT and DDLT. 
